# Application of Cs/GO/TiO_2_ as gas sensor

**DOI:** 10.1038/s41598-025-14525-8

**Published:** 2025-08-25

**Authors:** Amged G. El-Srougy, Khaled S. Amin, Mohamed M. Mahmoud, Mahmoud S. Ghanem, Hanan Elhaes, Medhat A. Ibrahim

**Affiliations:** 1 Physics Department, Faculty of Science, Mansoura University, Mansoura, Egypt; 2 Physics Department, Faculty of Science, Al-Azhar University, Cairo, Egypt; 3 Physics Department, Faculty of Women for Arts, Science and Education, Ain Shams University, Cairo, 11757 Egypt; 4 Spectroscopy Department, National Research Centre, 33 El-Bohouth St., 12622, Dokki, Giza, Egypt; 5 Molecular Modeling and Spectroscopy Laboratory, Centre of Excellence for Advanced Science, National Research Centre, 33 El-Bohouth St., 12622, Dokki, Giza, Egypt

**Keywords:** Chitosan, GO, DFT: B3LYP/LANL2DZ, Gas sensor, TiO_2_, Nanocomposite, QTAIM, Materials science, Physics

## Abstract

Chitosan (Cs), a biodegradable, and low-cost polymer is a good choice for gas sensor applications. In this study, Cs was modified with graphene oxide (GO) and titanium dioxide (TiO_2_), and its electronic properties were calculated using density functional theory (DFT) at B3LYP/LANL2DZ level. The calculated physical parameters include total dipole moment (TDM), HOMO/LUMO energy gap (ΔE), global reactivity descriptors and density of states (DOS) and mapping the electrostatic potential (MESP). Results indicated Cs had significant modification such as enhanced ΔE from 6.908 to 2.197 eV and TDM from 5.884 to 14.432 Debye, global reactivity revealed enhanced reactivity with increased absolute softness and high electrophilicity index. DOS show more available states and more localized HOMO/LUMO orbitals all enhance charge transfer. MESP shows reactivity and active sites for the interaction with its surroundings. The nanocomposite Cs/GO/TiO_2_ is supposed to interact with three different gases: H_2_O, CO_2_, and CH_4_. The results exhibited changes in the ΔE and TDM, with Cs/GO/TiO_2_/CO_2_ have the most pronounced changes. Partial density of states PDOS plots exhibited Ti atoms contribution in HOMO orbitals and LUMO with Cs/GO/TiO_2_/CO_2_ having the most changes in its energy states. Adsorption energy (E_a_) and Gibbs free energy (ΔG) calculations revealed that CH₄ (E_a_ = 4.396 eV, ΔG = − 3.684 eV) and H₂O (E_a_ = 4.000 eV, ΔG = − 3.263 eV) exhibited stronger and more favorable adsorption than CO₂ (E_a_ a = − 0.104 eV, ΔG = + 0.801 eV). Non-covalent interaction (NCI) and Quantum theory of atoms in molecules (QTAIM) confirmed the weak interaction with gases molecules and enhanced stability via hydrogen and vdWs bonding. The Cs/GO/TiO_2_ nanocomposite was synthesized, and FTIR spectroscopy was conducted and compared with calculated IR to verify the models.

## Introduction

 Biopolymers have garnered significant interest in sensor technology because of their environmentally friendly characteristics, availability, and straightforward processing^1^. Every year, around 100 billion tons of chitin is produced naturally on our planet by crustaceans, mollusks, insects, fungi, and similar organisms. At present, the extraction of chitin in industrial settings primarily depends on chemical techniques and the utilization of marine shell waste streams^2^. Chitosan (Cs), a random copolymer derived from chitin, demonstrates significant versatility. In acidic environments, the amino groups along its polymer chains become protonated, resulting in the polymer being cationic. This distinctive characteristic allows chitosan to engage with a wide variety of molecules, setting it apart as the sole cationic marine polysaccharide. The positive charge is thought to play a crucial role in its antimicrobial activity^3^, considering its plentiful availability and remarkable functional characteristics including biocompatibility, bioactivity, biodegradability, and impressive mechanical strength^2–4^. The field of gas sensing is essential for identifying dangerous gases, keeping an eye on air quality, and guaranteeing environmental safety. While traditional gas sensors often utilize materials like metal oxides and semiconductors, there is a growing demand for sustainable, cost-effective, and environmentally friendly alternatives^5^. Chitosan, with its excellent film-forming ability and hydrophilic nature, has emerged as a promising candidate for gas sensing applications. Chitosan’s gas-sensing capabilities can be further enhanced by incorporating other materials. For example, chitosan’s responsiveness and reproducibility in detecting acetone gas are much improved when combined with metal oxides, like SnO_2_, leading to larger output voltages and improved performance^6^. Similarly, hybrid films created by blending chitosan with conductive polymers like polypyrrole (Ppy) or nanoparticles such as zinc oxide (ZnO) exhibit improved electrochemical properties. These hybrid materials demonstrate high selectivity and responsiveness toward specific gases, such as hydrogen^7,8^. Chitosan’s abundance of hydroxyl (-OH) and amino (-NH_2_) groups further enhances its hydrophilicity. The adsorption of water molecules is facilitated by these functional groups, increasing its sensitivity to variations in humidity and bolstering its potential for advanced environmental monitoring applications^9^. The incorporation of graphene oxide (GO) can enhances the performance of chitosan by providing high surface area, good electrical conductivity, and high oxygen-containing functional groups. Synergism between chitosan and GO causes a substantial improvement in the electrical and sensing properties of the composite material. The incorporation of GO into chitosan not only changes the band gap but also enhances the charge transfer characteristics, which are extremely important for increased sensitivity and responsivity of the sensors^10,11^. In addition, GO/chitosan composites have been reported to greatly improve the mechanical stability and strength of the composite material and make the composite material stronger for diverse sensing purposes^12^. Furthermore, the oxygen-functional groups in GO enhance the interaction with target molecules, which makes sensors more sensitive and selective^13,14^. Titanium dioxide (TiO₂) is a prominent n-type semiconductor characterized by a band gap of around 3 eV. Its advantageous catalytic properties, durability, and environmental safety render it useful in a range of applications, such as gas sensors, solar cells, and photocatalytic processes^15,16^. TiO₂ is found in three primary crystalline forms: rutile, anatase, and brookite, with rutile being the most stable thermodynamically^17^. Gas sensors based on TiO₂ are extensively utilized for the detection of harmful gases, owing to their sensitivity, rapid response times, and affordability^18^. The incorporation of nanostructures significantly enhances the gas-sensing performance by increasing the surface-to-volume ratio, which facilitates the detection of various gases, including ozone, ethanol, acetone, hydrogen, and carbon monoxide^19–22^.

Molecular modeling is a class of computational work which has been recognized as a powerful approach to elucidate the electronic properties for many molecular systems^23,24^. It was reported that, the DFT could describe intramolecular charge transfer in fluoranthene derivatives^23^. It became useful in studying the behavior and mechanism of interaction for sensors^25,26^. For simple organic dyes molecule DFT supported different experimental findings including optical, electrochemical and thermal properties^24^. It was reported that, for gas sensing phenomena density functional theory DFT could support the experimental findings and became an important tool in this field^27^. DFT could be descriptive tool for mechanism of interaction between certain gas and a given molecular surface. For example, recent DFT research on PtnBe clusters showed that gas adsorption depends on cluster size, with strong chemisorption for most gases and rapid CH₄ recovery due to weak physisorption^28^.

The present work investigates the synthesis and modeling of a Cs/GO/TiO₂ utilizing DFT for enhancing of the possible applications of such nanocomposite. In this study, three gases were selected for analysis: CO₂, CH₄, and H₂O. CO₂ was chosen due to its environmental and health relevance as a greenhouse gas. CH₄ represents a commonly used flammable gas, significant for safety monitoring in homes, laboratories, and industrial settings, particularly in Egypt. H₂O was included as a representative of humidity, which is especially important in controlled environments like museums and archives. These choices reflect a range of practical sensing targets: toxic gases, fuel gases, and moisture. DFT at B3LYP/LANL2DZ level was applied to evaluate physical parameters including the total dipole moment (TDM), the HOMO–LUMO and energy gap (ΔE). The density of states (DOS), HOMO-LUMO frontier orbitals, global reactivity descriptors, and molecular electrostatic potential (MESP) will clarify the active sites and charge transfer mechanisms involved in gas interactions. Moreover, Quantum theory of atoms in molecules (QTAIM) was conducted to investigate non-covalent interactions, and the adsorption energies to investigate selectivity. Finally, a comparison between theoretical predictions and Fourier-transform infrared (FTIR) spectra was conducted to confirm the computational models. This computational strategy aims to create advanced nanocomposite that combine polymeric materials and metal oxides, utilizing the advantages of Cs, GO, and TiO_2_ which enhance their properties, especially for gas sensing applications with improved selectivity and sensitivity.

## Materials and methods

### Calculation details

The models have been investigated using the G09 program^29^. The structures were computed at Molecular Modeling and Spectroscopy Laboratory, Centre for Excellence for Advanced Science, National Research. For the structural optimizations calculations, the B3LYP functional (which combines Becke’s three-parameter exchange functional with the Lee-Yang-Parr correlation functional) were employed uniformly across all atoms in this study to ensure consistency in the computational treatment of the Cs/GO/TiO₂ nanocomposite^30–32^. While we acknowledge that LANL2DZ^33^ is not the most accurate basis set for lighter elements, its application here provides a consistent framework for comparing electronic trends, utilizing consistent computational methodology. LANL2DZ was used exclusively for Ti, and that the D95V^34^ basis set was employed for the lighter atoms (C, H, O, N). Upon optimized structures the total dipole moment (TDM), highest occupied molecular orbital (HOMO), and lowest unoccupied molecular orbital (LUMO) were calculated. The energy gap (∆E), derived from HOMO-LUMO energy difference. TDM and ∆E provides critical insights into molecular interaction potential and reactivity. The molecular electrostatic potential (MESP) allows for the visualization of the active sites. The Density of States (DOS) was graphically represented to elucidate electronic behavior. Global reactivity descriptors, including fundamental quantum chemical parameters such as Ionization Potential (IE), Electronic Affinity (A), Chemical Potential (µ), Chemical Hardness (η), Absolute Softness (S), and Electrophilicity Index (ω)^35–37^, were systematically calculated using the following formulas:

1 $$\:A=-{E}_{LUMO}$$

2 $$\:\mu\:=-\frac{IE\:+A}{2}$$

3 $$\:\eta\:=\frac{IE-A}{2}$$

4 $$\:S=\frac{1}{\eta\:}$$

5 $$\:{\upomega\:}=\frac{{\mu\:}^{2}}{2\eta\:}$$

6 $$\:IE=-{E}_{HOMO}$$

The adsorption energy E_a_ and for studied nanocomposites interacting with the adsorbed gases was calculated by the equation^38^:

7 $$E_a = - [E_{system} - (E_{adsorbent} + E_{adsorbate})]$$

To evaluate the spontaneity of gas adsorption, the Gibbs free energy of adsorption (ΔG°a_d_s) was computed using thermochemical data extracted from frequency calculations at 298 K. The total free energy was calculated as the sum of the electronic energy (E₀) and the thermal correction to Gibbs free energy (G_corr), as provided by the Gaussian 09 output. The expression used is:

8 $$\Delta {\text{r}}{\text{G}}{^\circ _{{\text{ads}}}}({\text{298K}}) = {({{\text{E}}_0} + {{\text{G}}_{{\text{corr}}}})_{{\text{adsorbed}}}}_{~{\text{complex}}} - [{({{\text{E}}_0} + {{\text{G}}_{{\text{corr}}}})_{{\text{adsorbent}}}} + {({{\text{E}}_0} + {{\text{G}}_{{\text{corr}}}})_{{\text{adsorbate}}}}]$$

QTAIM topology analyses were conducted with the use of Multiwfn and visual molecular dynamics (VMD) software^39,40^, to investigate the interaction with the adsorbed gases and the nature of the bonds formed as well as the stability of the nanocomposites. The calculated NCI was also generated by the same software^39,40^.Finally, frequency calculations were performed to confirm that the structures correspond to true minima and to compare them with the FTIR data to validate the DFT level of methodology. It is worth to mention that,

### Chemicals and reagents

Chitosan deacetylation degree 90% ±5 purchased from Chitosan Egypt LLC. Sulfuric acid (96%) was obtained from Scharlau and hydrogen peroxide from PIOCHEM (30%). Sodium hydroxide and ethanol were purchased from El Nasr Pharmaceutical Chemicals Co., Cairo, Egypt. Titanium tetraisopropoxide Ti (OC_3_H_7_)_4_ termed as TTIP was purchased from Sigma Aldrich – Germany. Ethyl alcohol (C_2_H_5_OH), Polyethylene glycol PEG (M.W 6000), and Acetic acid (CH_3_COOH) were purchased from El-Nasr Pharmaceutical Company, Egypt. Citric acid (99.5%), and sodium hydroxide (≥ 97%), phosphoric acid (85%), potassium permanganate (99%), and graphite powder were obtained from Fisher Chemical. Distilled water and deionized (DI) Milli-Q water was used during the preparations of samples.

### Preparation of TiO_2_

Nano titanium dioxide TiO_2_ synthesis was carried out using precipitation technique. By dissolving titanium tetraisopropoxide into absolute ethanol (in ice bath), while stirring at low base, water was added to the solution. To control the grain growth due to hydrolysis process, drops from Acetic acid and polyethylene glycol were added to the solution while stirred at high speed for 2 h. The solution then left over night for precipitation to occur. Then two layers formed, the upper layer was the organic byproducts of the hydrolysis, and the lower layer was a TiO_2_ precipitate. After that the TiO_2_ precipitate was filtered and washed several times with distilled water, then dried overnight at 100 °C. The yellow crystal blocks obtained were grounded into fine powder then calcinated for 3 h at 500 °C to get pure anatase nanoparticle of TiO_2_.

### Preparation of GO

The modified Hummer’s method was used to synthesize GO. 3 g of graphite flakes were mixed with 360 ml of sulfuric acid and 40 ml of phosphoric acid, the mixture is in a 9:1 ration and is stirred continuously. The mixture was cooled in an ice bath, and then 18 g of KMnO₄ was slowly added. The color of the mixture changes from black (graphite color) to a dark olive green, while temperature was regulated between 0 and 5 °C to minimize exothermic reactions. After stirring the mixture at 40 °C overnight, the mixture was mixed with 400 ml of iced deionized water containing 30% of H₂O₂. The color changed from buffer violet to light brown after this step. The mixture was filtered with centrifugation at 10,000 rpm and washed discarding the supernatant. The filtrate obtained was dried at 70 °C for five hours using an oven.

### Preparation of Cs/GO/TiO_2_

Cs was mixed with GO and TiO₂ to form a nanocomposite. Specifically, 0.25 g of Cs was dissolved in 50 mL of deionized water (2% acetic acid) under continuous stirring. Two samples were prepared, pure Cs of 0.250 g (100%) and a mixture containing 0.225 g of Cs (90%), 0.0125 g of GO (5%), and 0.0125 g of TiO₂ (5%). The samples were stirred for 30 min at 70 °C. The Cs/GO/TiO_2_ mixture were followed by 2–3 min of sonication to ensure uniform dispersion of GO within the nanocomposite. The resulting solutions were then drop-cast onto petri dishes and left to dry at room temperature for five days to form the final composite film.

### FTIR measurements

FTIR spectra were collected with the help of the Attenuated Total Reflection Fourier Transform Infrared (ATR-FTIR) using an FTIR spectrometer (Vertex 70, Bruker), which have a spectral range of 4000–400 cm^−1^ and a spectral resolution of 4 cm^−1^.

## Results and discussion

### Building module molecules

**Fig. 1:**
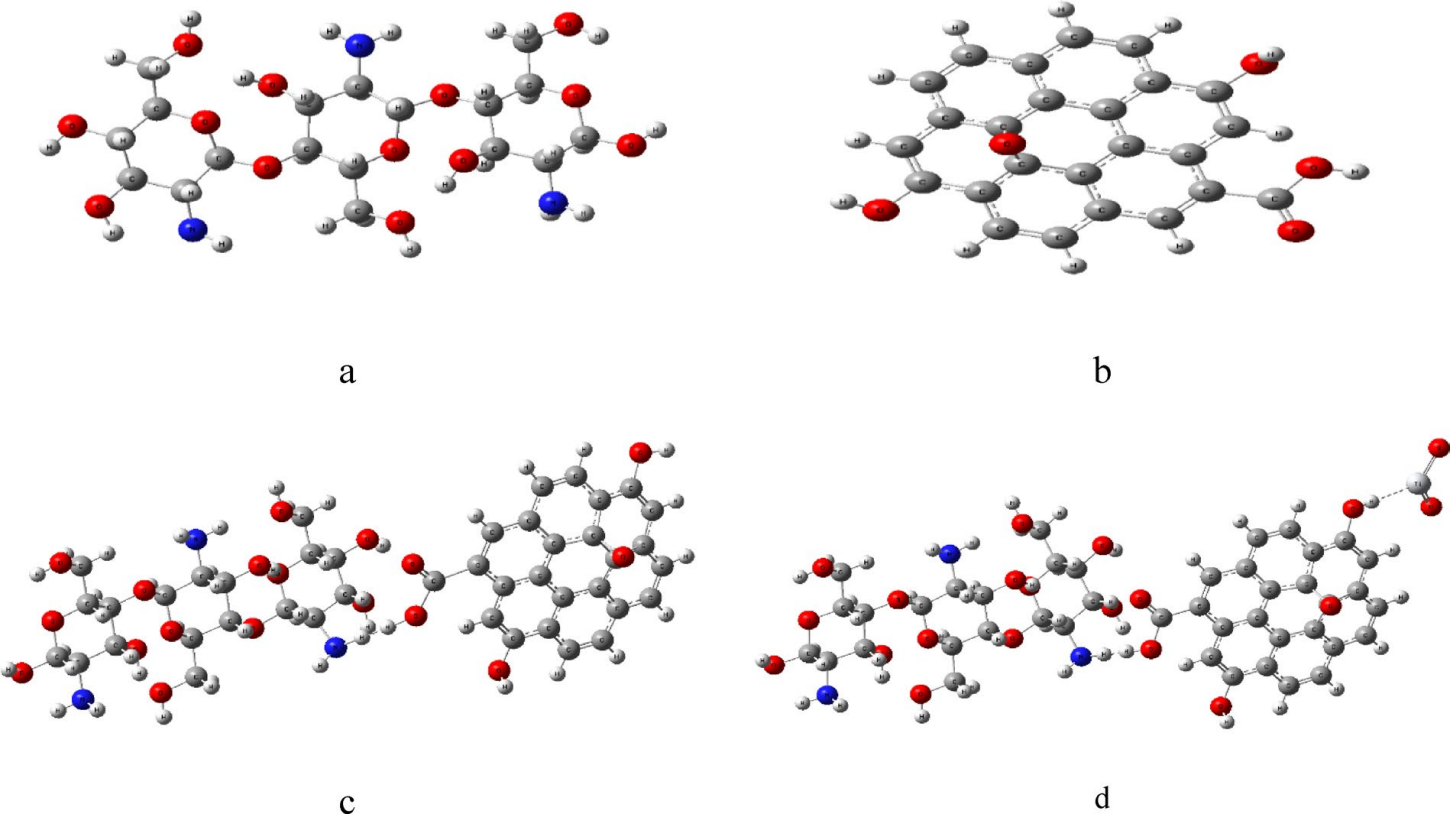
Module molecules structures for **a**- Chitosan (Cs), **b**- Graphene oxide (GO), **c**- Cs/GO and d- Cs/GO/TiO_2_. Figures were designed and implemented with Gauss View 5.0 software^41^.

Before commencing the DFT calculations, we constructed molecular models of the structures. Figures (1) displays the model structures where figure (1-a) shows three units of chitosan (Cs). Figures (1-b) shows Graphene oxide (GO) like structure with COOH, OH functional groups. Figures (1-c) shows Cs interacting weakly with GO through H atom of COOH and amine group of the Cs. Figures (1-d) the Cs/GO interacted weakly with TiO_2_ through terminal OH of GO via Ti atom of TiO_2_.

### Calculated physical parameters

The TDM provides valuable information about electronic charge distribution in molecules, hence has a crucial role in the establishment of molecular polarity. Such polarity notably influences the chemical properties and reactivity of the molecules as well as their interaction with the molecules that surround them. Elevated values of TDM may suggest that the structure exhibits heightened reactivity and enhanced capacity for interaction with surrounding molecules^42^. The HOMO–LUMO gap (ΔE) represents the energy difference between the highest occupied molecular orbital (HOMO) and the lowest unoccupied molecular orbital (LUMO). ΔE helps in understanding a molecule’s electronic properties and charge transport phenomena, providing insights into its stability and reactivity. Moreover, ΔE helps in understanding the electronic transport characteristics of molecular systems^43,44^. TDM and ΔE were calculated for the studied models using B3LYB/LANL2DZ method; the results are recorded in Table (1). In the case of Cs, TDM and ΔE were found to be 5.884 Debye and 6.908 eV respectively. When Cs interacted with GO the TDM decreased a little to become 5.489 Debye but the ΔE significantly decreased to 2.748 eV. The addition of TiO_2_ further improved the ΔE values to reach 2.197 eV and enhanced the TDM which reached 14.432 Debye. This means that the composite Cs/GO/TiO_2_ is more reactive than Cs or Cs/GO alone.

**Table 1:** Calculated TDM in Debye and ΔE in eV for the studied structures.

Structures	TDM (Debye)	ΔE (eV)
**Cs**	5.884	6.908
**GO**	3.861	2.758
**Cs/GO**	5.489	2.748
**Cs/GO/TiO**_**2**_	14.432	2.197

### Global reactivity descriptors

HOMO and LUMO values were calculated using B3LYB/LANL2DZ method, and Global reactivity IE, A, µ, η, S and ω can be defined and computed by the equations (1:6). The calculated global reactivity descriptors for Cs, GO, Cs/GO and Cs/GO/TiO_2_ composite were recorded in Table (2). Ionization energy (IE) value for the composite Cs/GO/TiO_2_ shows lower value than Cs (from 5.931 to 5.614 eV). This means that lower energy is required to remove an electron from the surface of the structure. Electron affinity (A) of Cs increased significantly from − 0.977 to 3.147 eV as Cs interacted with GO/TiO_2_ which indicates more energy when electron added to the system^35^. Hardness (η) and softness (S) values can be described as an indication of the inhibition of inter-molecule transfer of charge. The composite Cs/GO/TiO_2_ displays much lower value for hardness (η) (1.099 eV) compared with the pure Cs (3.454). While softness (S) value of pure Cs displayed the lower value (0.290 eV^−1^) than the composite Cs/GO/TiO_2_ (0.910 eV^−1^) which indicate more reactivity of the composite Cs/GO/TiO_2_. The composite also shows higher values for µ and ω compering with pure Cs, which indicate that the composite structure has a tendency to accept electrons and have better electrophilic characteristics.

**Table 2:** Calculated global reactivity descriptors for the studied structures.

Structure	IE	A	µ	η	S	ω
**Cs**	5.931	−0.977	−2.477	3.454	0.290	0.888
**GO**	5.426	2.669	−4.048	1.379	0.725	5.941
**Cs/GO**	5.370	2.622	−3.996	1.374	0.728	5.812
**Cs/GO/TiO**_**2**_	5.614	3.417	−4.515	1.099	0.910	9.279

### Mapping molecular electrostatic potential MESP

**Fig. 2:**
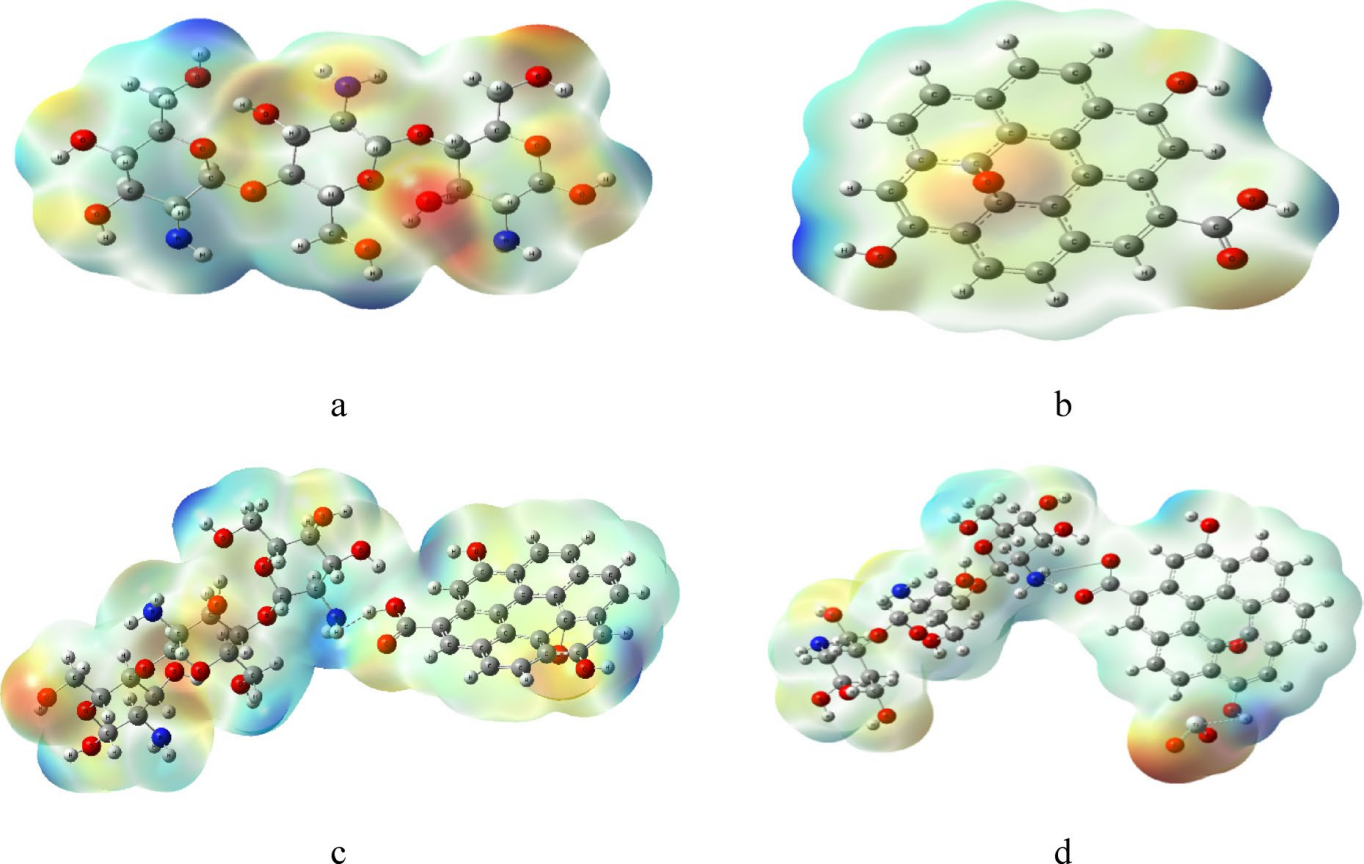
Calculated MESP for the modeled structure where **a**- Cs, **b**- GO, **c**- Cs/GO, and **d**- Cs/GO/TiO_2_. Figures were implemented with Gauss View 5.0 software^41^.

The molecular electrostatic potential MESP were calculated using B3LYB/LANL2DZ method. MESP can show the reactivity of molecules by a color code and show the active sites^45^. The color ranges from red to blue where red represents negative regions and blue is positive region of electrostatic potential, while green color is more neutral. Figures (2) shows the calculated MESP for the studied structures. In figure (2-a), MESP of pure Cs shows red regions around the O atoms and blue regions around the H atom of OH. For Cs/GO/TiO_2_ nanocomposite (figure (2-d)), the structure shows more red region around the O atoms of TiO_2_ indicating a more reactive structure to surrounding molecules^46^.

### Frontier molecular orbitals

**Fig. 3:**
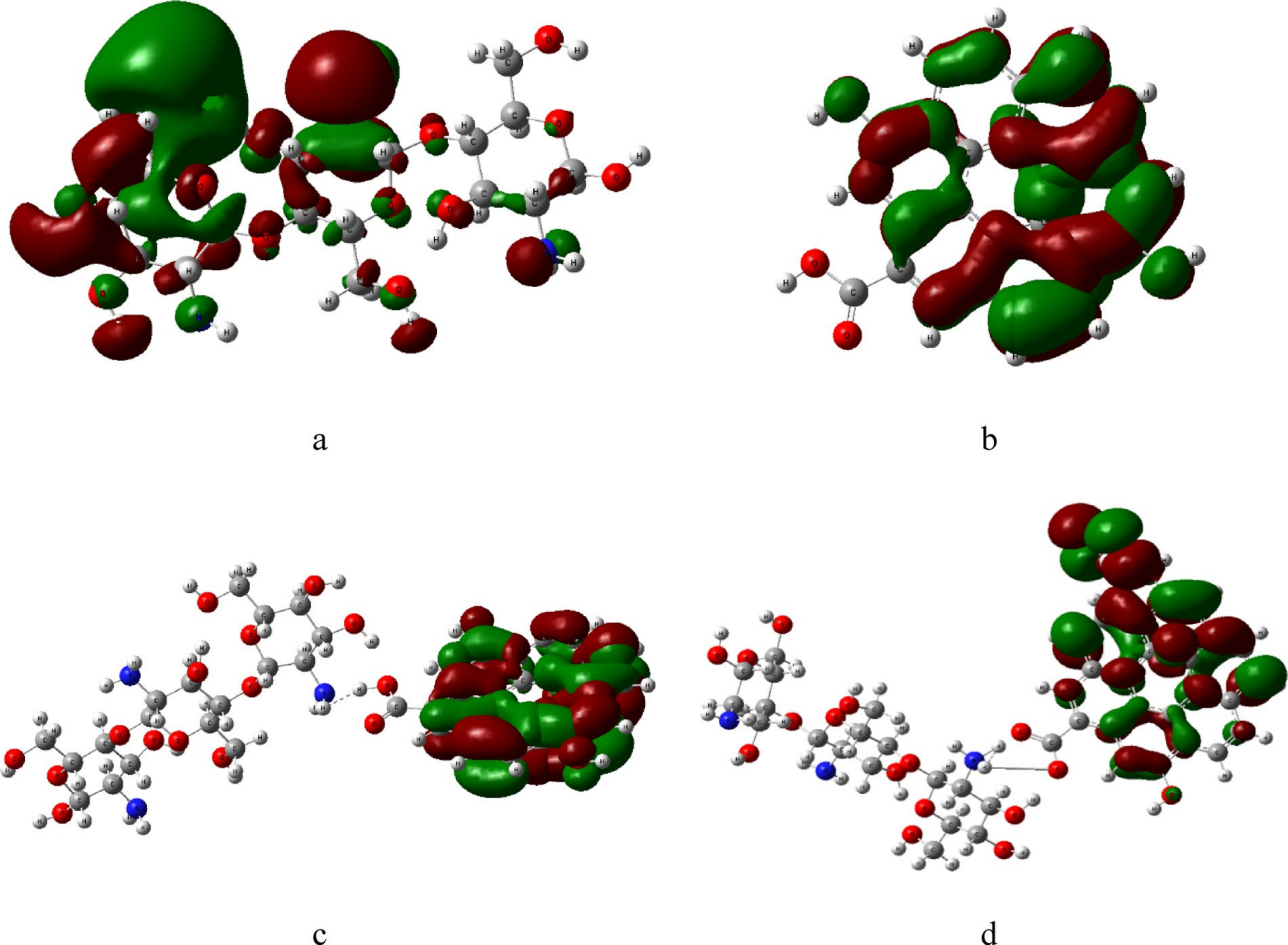
Calculated HOMO/LUMO for the modeled structure where a- Cs, b- GO, c- Cs/GO and d- Cs/GO/TiO_2_. Figures were implemented with Gauss View 5.0 software^41^.

The HOMO/LUMO frontier molecular orbitals were calculated using B3LYB/LANL2DZ method. HOMO and LUMO are the most essential parameters in quantum chemistry^47^. In chemistry, HOMO and LUMO are types of molecular orbitals. The acronyms stand for highest occupied molecular orbital and lowest unoccupied molecular orbital, respectively, and the difference between them is called the HOMO–LUMO gap. The calculation of HOMO and LUMO gives information on the transfer of charge within the molecule^48^. Figures (3) presents the distribution of HOMO and LUMO frontier orbitals where the red color denotes the positive phase, and the green color denotes to the negative phase. The HOMO–LUMO map of pure Cs and GO molecules are shown in figure (3-a) and (3-b) respectively. When Cs interacts with GO as shown in figure (3-c) all the electronic charge activity, HOMO-LUMO, shifted to the GO molecule. When TiO_2_ molecule is added to the Cs/GO structure as shown in figure (3-d), HOMO localized on TiO_2_ and the LUMO on GO this localization enhance charge transfer interactions^49^. This indicates enhancement in charge transfer and electron mobility.

### Density of States DOS

**Fig. 4:**
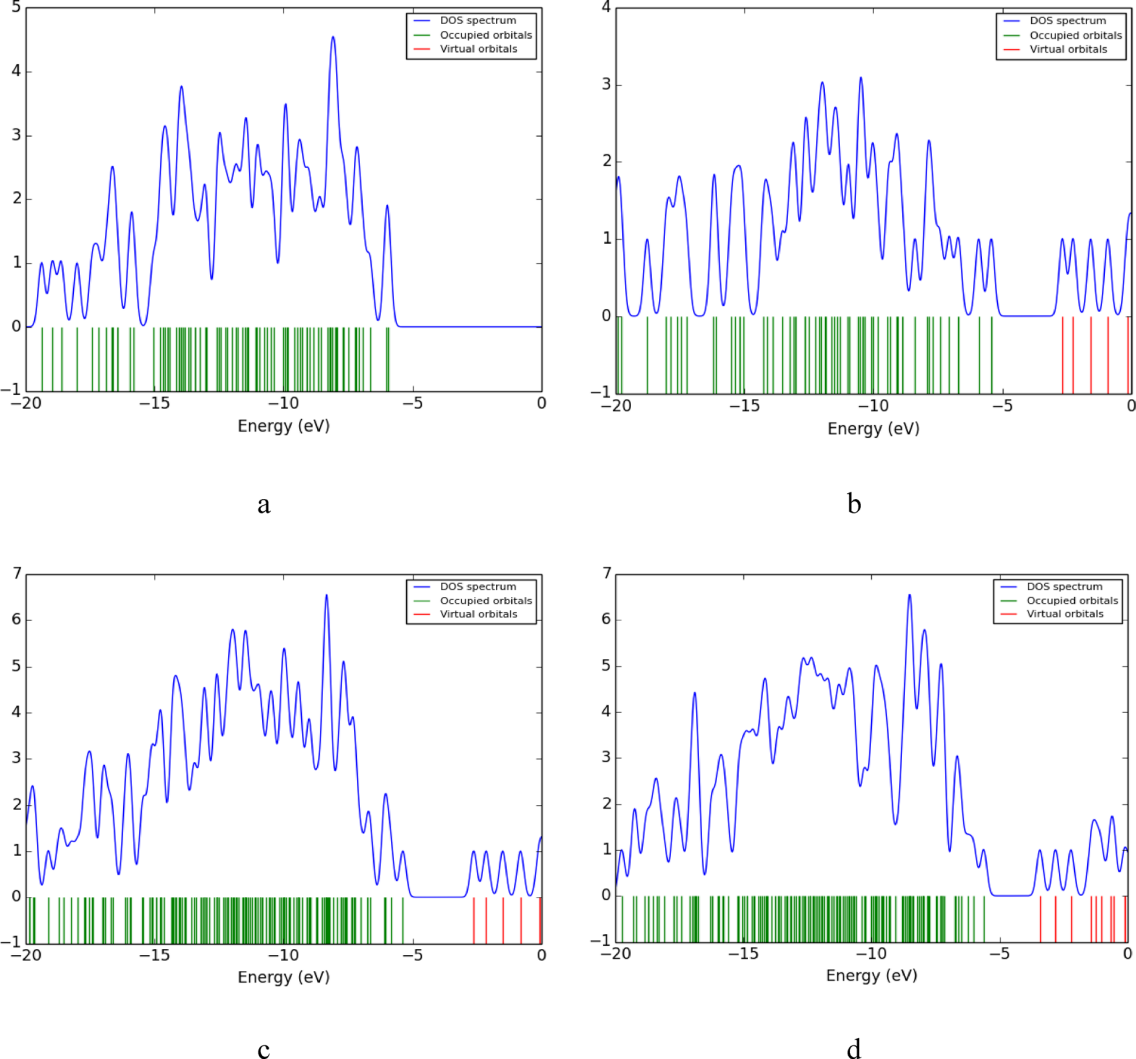
Calculated DOS for the modeled structure where **a**- Cs, **b**- GO, **c**- Cs/GO, and **d**- Cs/GO/TiO_2_.

The density of states (DOS) for the studied structures calculated using DFT: B3LYB/LANL2DZ method is displayed in figure (4). DOS represents the number of states per energy level, where the green lines represent occupied orbitals and the red lines correspond to the unoccupied (virtual) orbitals. In figure (4-c) the nanocomposite Cs/GO exhibits a decrease in bandgap compared to Cs alone (figure (4-a)). After the incorporation of TiO_2_ the bandgap decreased further, and the number available unoccupied stated near the Fermi level increased as shown in figure (4-d). This enhancement in available states facilitates charge transfer, as evidenced by the shifted virtual orbitals toward the Fermi level.

### Gas sensing application

**Fig. 5:**
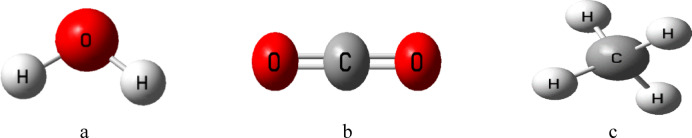
Module gas molecules structures whereas **a**- H_2_O, **b**- CO_2_ and **c**- CH_4_.

**Fig. 6:**
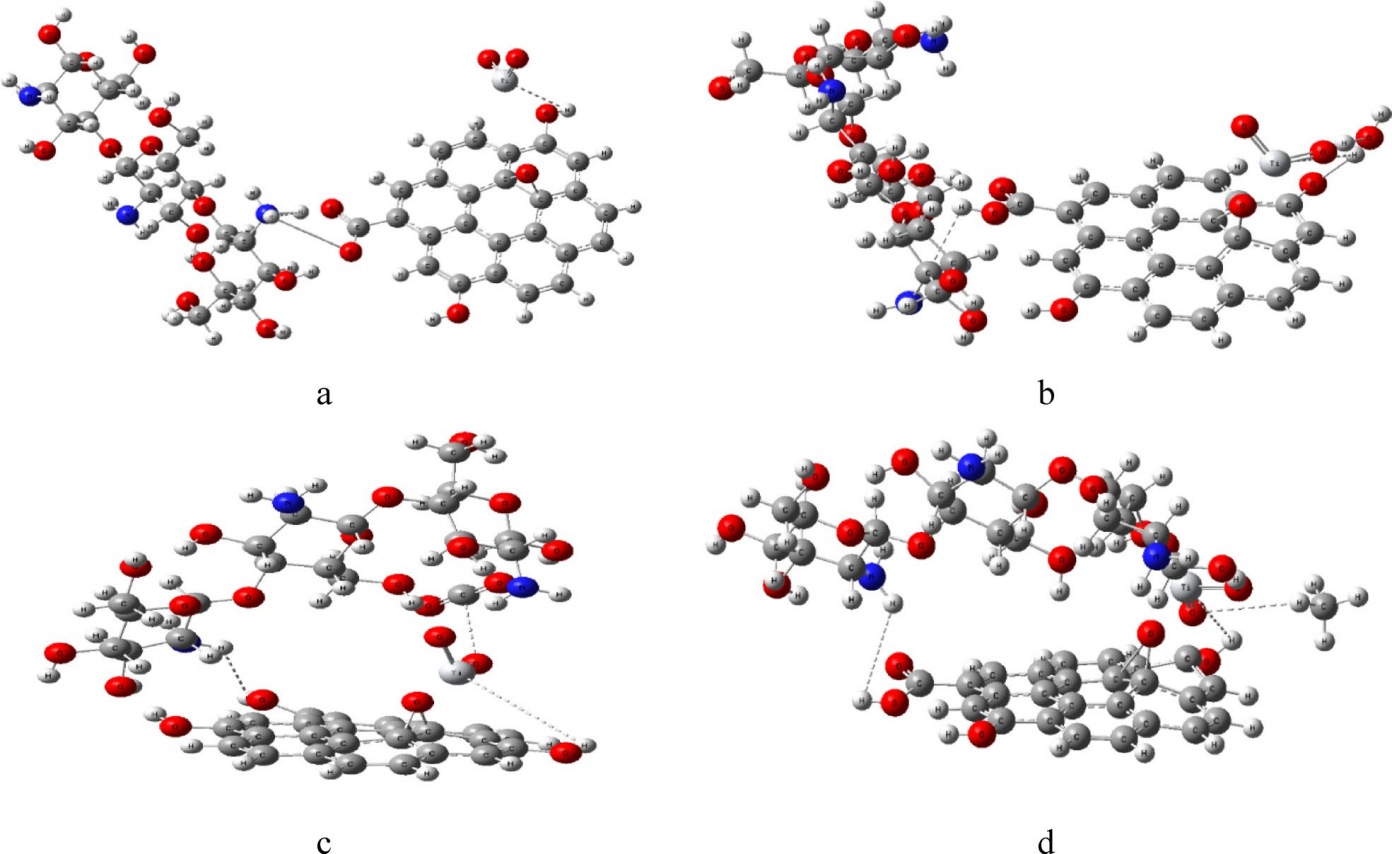
The physical interaction between Cs/GO/TiO_2_ nanocomposite with the gas molecules, where; **a**- Cs/GO/TiO_2_, **b**- Cs/GO/TiO_2_/H_2_O, **c**- Cs/GO/TiO_2_/CO_2_ and **d**- Cs/GO/TiO_2_/CH_4_. Figures were implemented with Gauss View 5.0 software^41^.

The Cs/GO/TiO_2_ nanocomposite was proposed to interact with three different gasses H_2_O, CO_2_ and CH_4_, the gases models can be shown in figure (5). The structures were optimized at the same computational level. The optimized structures are recorded in figure (6), where figure (6-a) presents Cs/GO/TiO_2_ nanocomposite, figure (6-b) presents Cs/GO/TiO_2_ interacting with H_2_O, figure (6-c) presents Cs/GO/TiO_2_ interacting with CO_2_ and figure (6-d) presents Cs/GO/TiO_2_ interacting with CH_4_. In these models, the O atom of TiO_2_ was supposed to interact weakly with the gas’s molecules. Electronic properties including total dipole moment (TDM) and HOMO-LUMO energy gap (ΔE) were calculated to quantify changes induced by gas adsorption.

Table (3) displays the changes in TDM and ΔE values of the nanocomposite upon interaction with H_2_O, CO_2_, and CH_4_ gases. Compared to the pristine nanocomposite TDM and ΔE values (14.432 Debye and 2.197 eV), significant changes in both parameters were observed upon interaction with H_2_O, CO_2_, and CH_4_ gases. This indicates a sensitivity of the nanocomposite to these gases. The largest variation was observed with CO_2_ interaction, where TDM increased from 14.432 Debye to 22.292 Debye, and ΔE decreased from 2.197 eV to 1.661 eV. This change in pristine nanocomposite values suggests strong interaction and high sensitivity towards CO_2_ sensing. In contrast, the CH_4_ interaction demonstrated the minimum change in TDM, which decreased to 7.887 Debye, and ΔE increased to 3.163 eV. The interaction with H_2_O shows moderate changes with TDM decreasing to 9.411 Debye and ΔE increasing to 3.958 eV.

**Table 3:** Calculated TDM in Debye and ΔE in eV for the studied structures.

Structures	TDM (Debye)	ΔE (eV)
**Cs/GO/TiO**_**2**_	14.432	2.197
**Cs/GO/TiO**_**2**_ **/H**_**2**_ **O**	9.411	3.958
**Cs/GO/TiO**_**2**_ **/CO**_**2**_	22.292	1.661
**Cs/GO/TiO**_**2**_ **/CH**_**4**_	7.887	3.163

**Fig. 7:**
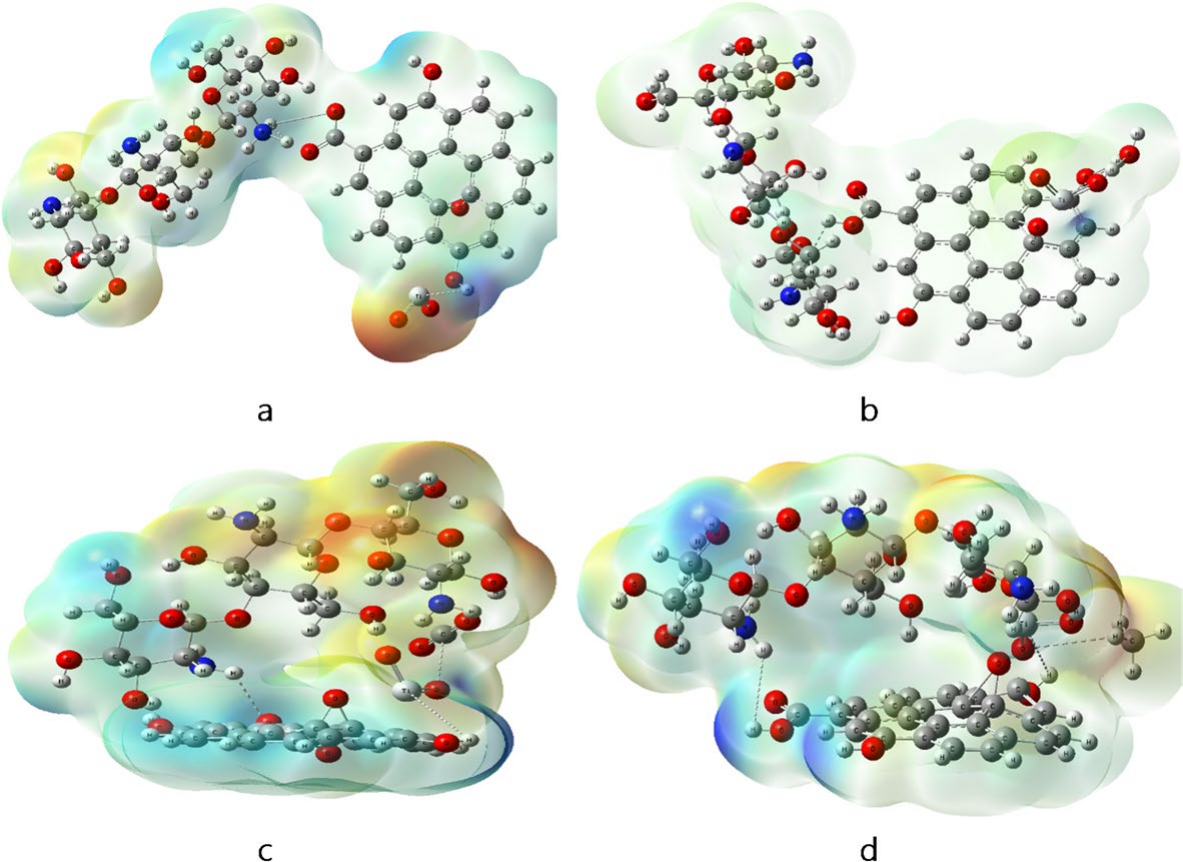
Calculated MESP for Cs/GO/TiO_2_ interacted with gas molecules, where; **a**- Cs/GO/TiO_2_, **b**- Cs/GO/TiO_2_/H_2_O, **c**- Cs/GO/TiO_2_/CO_2_ and **d**- Cs/GO/TiO_2_/CH_4_. Figures were implemented with Gauss View 5.0 software^41^.

MESP were calculated for Cs/GO/TiO_2_ interacted with gas molecules and some changes in the charge distribution occurred, due to the interaction. The red regions around O atoms of TiO_2_ in figure (7-a) disappeared for Cs/GO/TiO_2_/H_2_O and a neutral surface is dominant (figure (7-b)). The Cs/GO/TiO_2_ interacting with CO_2_ in figure (7-c), shows more spread reactive regions (red and blue) which indicate that the nanocomposite is susceptible to more interaction with gas molecules. The Cs/GO/TiO_2_ interacting with CH_4_ exhibit less reactivity as shown in figure (7-d). These MESP changes align with observed trends in TDM and ΔE modulation.

**Fig. 8:**
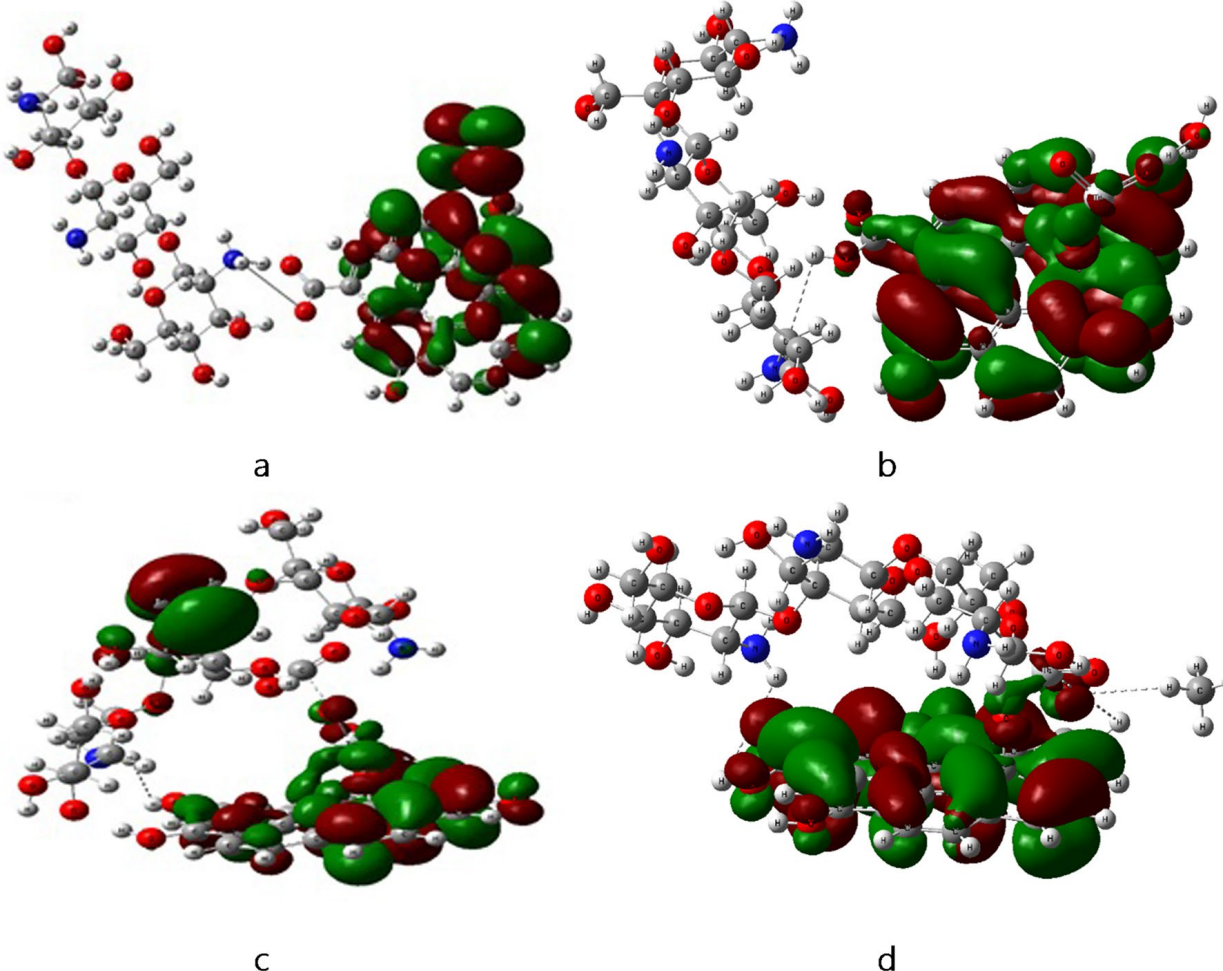
Calculated HOMO/LUMO for Cs/GO/TiO_2_ interacted with gas molecules, where; **a**- Cs/GO/TiO_2_, **b**- Cs/GO/TiO_2_/H_2_O,** c**- Cs/GO/TiO_2_/CO_2_ and **d**- Cs/GO/TiO_2_/CH_4_. Figures were implemented with Gauss View 5.0 software^41^.

The HOMO-LUMO frontier orbitals of Cs/GO/TiO₂ were analyzed post-interaction with gases. In the pristine nanocomposite (figure (8-a)), the HOMO localizes on TiO₂, while the LUMO resides on GO. Interaction with H₂O (figure (8-b)) shifts the HOMO to GO and distributes the LUMO across both GO and TiO₂. For CH₄ adsorption in figure (8-d) the HOMO and LUMO are distributed on GO and TiO_2_. For CO₂ adsorption (figure (8-c)), the HOMO occupies the amine group of Cs, and the LUMO remains on GO/TiO₂. These orbital redistributions highlight analyte-specific electronic modulation, aligning with observed TDM and ΔE trends.

### Partial density of States (PDOS)

**Fig. 9:**
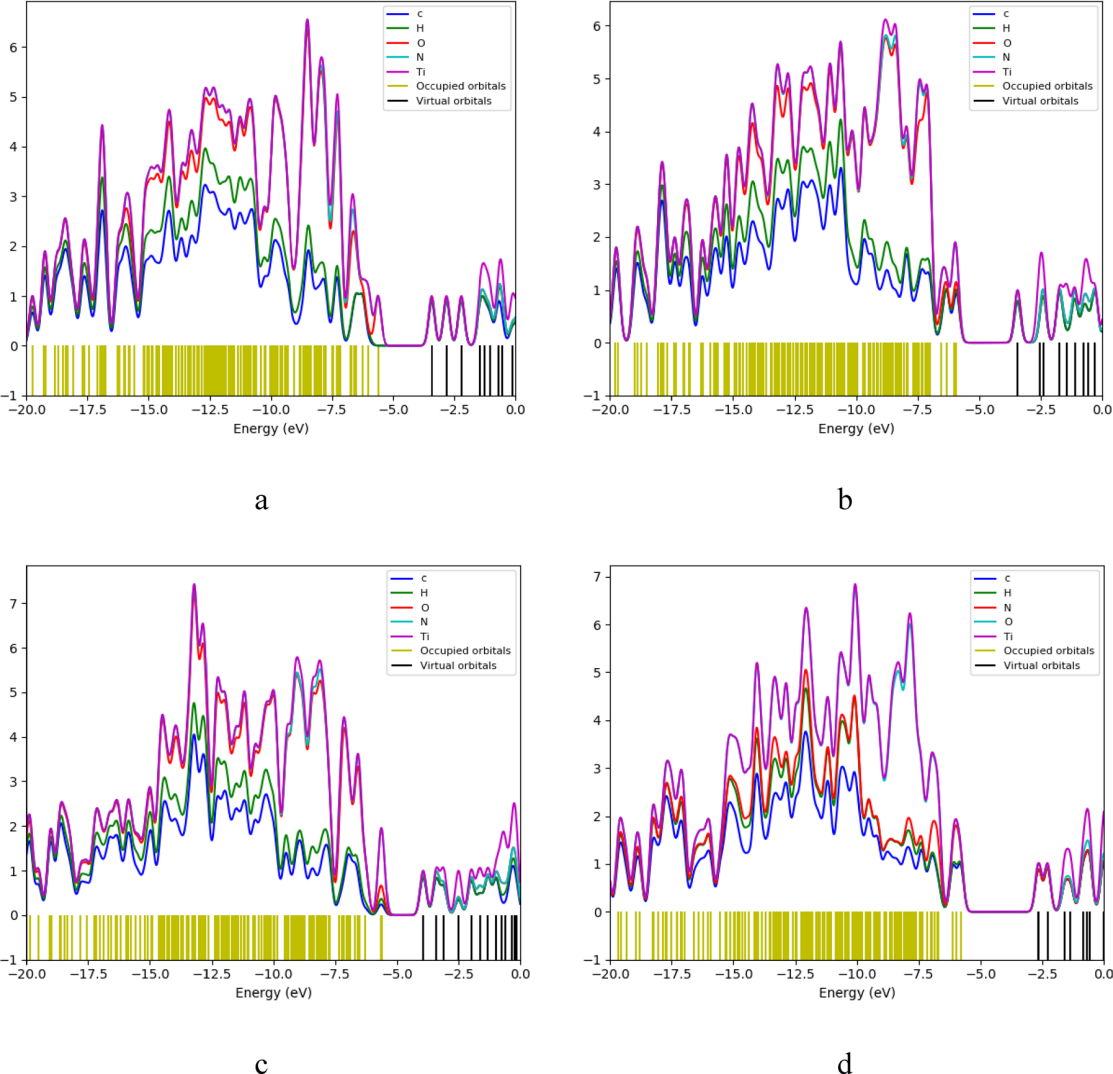
Calculated PDOS for Cs/GO/TiO_2_ nanocomposite and the nanocomposite interacting with gas molecules, where; **a**- Cs/GO/TiO_2_ **b**- Cs/GO/TiO_2_/H_2_O, **c**- Cs/GO/TiO_2_/CO_2_ and **d**- Cs/GO/TiO_2_/CH_4_.

The projection of a specific atom’s orbital on the density of states is provided by PDOS, where the total density of state (DOS), can be obtained by adding up all the projections. PDOS plots for Cs/GO/TiO_2_ nanocomposite and its interaction with the gases are displayed in figure (9). The Ti atom contributes the most in both the unoccupied (virtual) and occupied orbitals, followed by O atoms. PDOS plot for Cs/GO/TiO_2_ is shown in figure (9-a), there are some changes in DOS upon interaction with the gases. Figures (9-c) represents PDOS for Cs/GO/TiO_2_ interacting with CO_2_, the changes in the states with the introduction of new states in the unoccupied orbitals (black lines), this makes charge transfer easier to occur. The changes in the DOS are not as potent for the other gases as shown in figure (9-b) and figure (9-d).

### Charge distribution

**Fig. 10:**
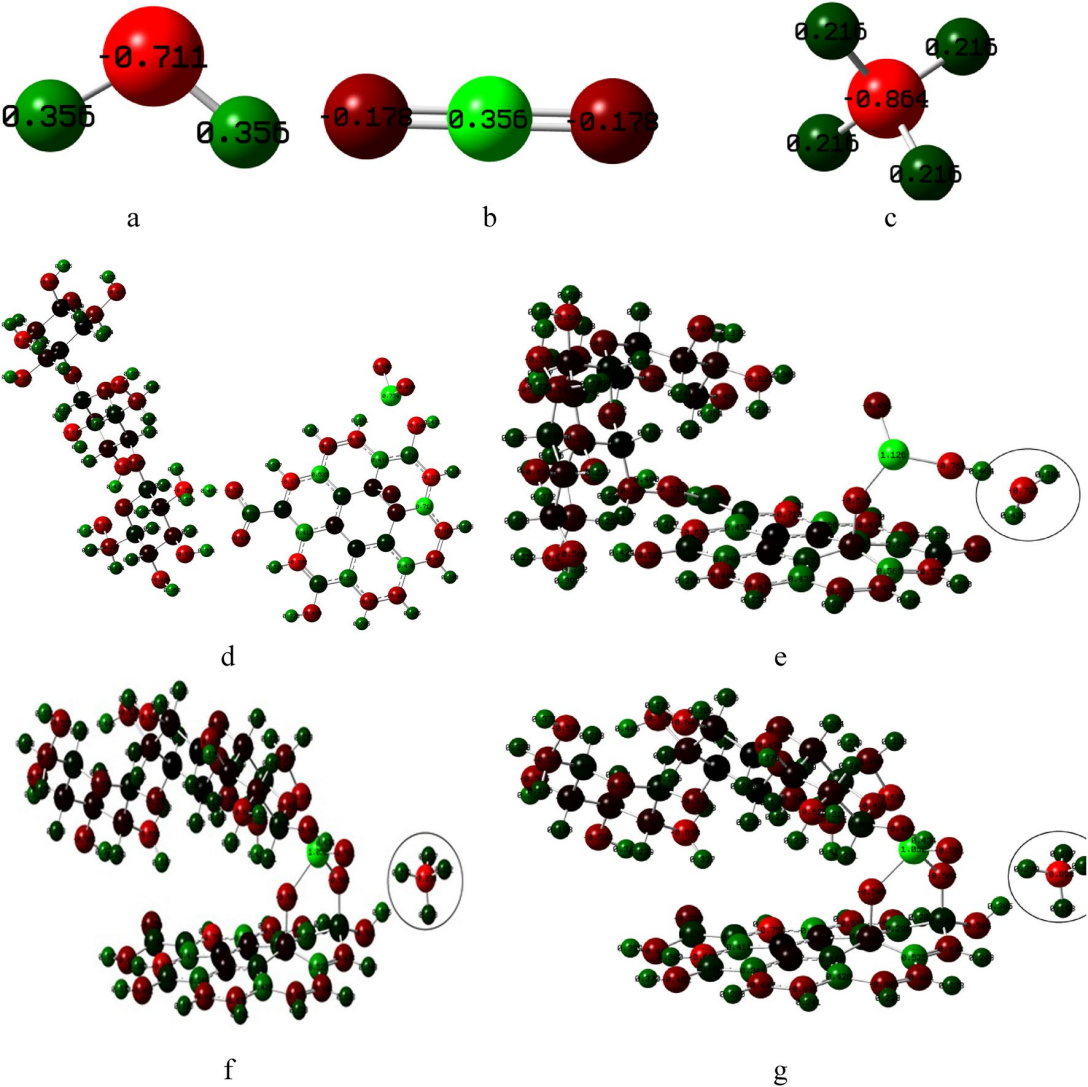
Mullikan population for charge distribution for Cs/GO/TiO_2_ and its interaction with gas molecules, where; **a**- H_2_O, **b**- CO_2_ and **c**- CH_4_, **d**- Cs/GO/TiO_2_, **e**- Cs/GO/TiO_2_/H_2_O, **f**- Cs/GO/TiO_2_/CO_2_ and **g**- Cs/GO/TiO_2_/CH_4_. Figures were implemented with Gauss View 5.0 software^41^.

Mulliken population analysis was performed at the same level of theory for the isolated gas molecules, the Cs/GO/TiO₂ nanocomposite, and their respective adsorption complexes. The charge distributions for the studied structures are presented in figure (10). As shown, noticeable changes in partial charges occur upon gas adsorption—particularly in the gas molecules (Fig. 10- a: c), which exhibit slight shifts in electron density following interaction with the composite. Additionally, charge redistribution is observed around the Ti atom in the nanocomposite. These results are consistent with the trends observed in the HOMO–LUMO energies, DOS, and MESP analyses, further supporting the presence of weak but meaningful electronic interactions between the gases and the sensing material.

### Adsorption energy and Gibbs free energy

To investigate the selectivity of Cs/GO/TiO_2_ nanocomposite for the studied gases, adsorption energy E_a_ were calculated, the results are recorded in Table (4). If E_a_ is negative value that means the interaction needs energy to occur and the interaction is endothermic. In contrast, if E_a_ is positive value that indicates the interaction is favorable. Adsorption energy results showed significant selectivity for CH_4_ and H_2_O adsorption where E_a_ have a value of 4.396 eV and 4.000 eV, for the adsorption onto Cs/GO/TiO_2_ nanocomposite. While E_a_ of the adsorption with CO_2_ gases onto Cs/GO/TiO_2_ nanocomposite were found to be −0.104. For comparison, a previous DFT study reported an E_a_ of **−** 0.590 eV for CH₄ adsorption on GO-epoxy surfaces alone, implying that the inclusion of TiO₂ and chitosan in our composite significantly enhances methane binding^50^.

To assess the spontaneity and thermodynamic feasibility of these interactions, Gibbs free energy (ΔG) of adsorption was conducted using thermochemical corrections from frequency calculations at 298 K. As recorded in Table 5, CH₄ and H₂O adsorption are exothermic and spontaneous, with ΔG values of − 3.684 eV and − 3.263 eV, respectively. In contrast, CO₂ was found to be non-spontaneous, with a ΔG of + 0.801 eV. Notably, these values compare favorably with reported Gibbs free energy values for CH₄ activation on anatase (+ 0.49 eV) and rutile TiO₂ (− 1.13 eV)^51^, likely due to synergistic effects from combining chitosan, GO, and TiO₂ that enhance adsorption under ambient conditions.

**Table 4:** Calculated adsorption energy E_a_ as eV for the studied gases onto Cs/GO/TiO_2_ nanocomposite.

Structure		Adsorption energy (eV)
**Cs/GO/TiO**_**2**_ **/H**_**2**_ **O**		4.000
**Cs/GO/TiO**_**2**_ **/CO**_**2**_		−0.104
**Cs/GO/TiO**_**2**_ **/CH**_**4**_		4.396

**Table 5:** Calculated Gibbs free energy of adsorption (ΔG°a_d_s) as eV for the studied gases onto Cs/GO/TiO_2_ nanocomposite.

Structure		ΔG (eV)
**Cs/GO/TiO**_**2**_ **/H**_**2**_ **O**		−3.263
**Cs/GO/TiO**_**2**_ **/CO**_**2**_		0.801
**Cs/GO/TiO**_**2**_ **/CH**_**4**_		−3.684

### QTAIM analyses

**Fig. 11:**
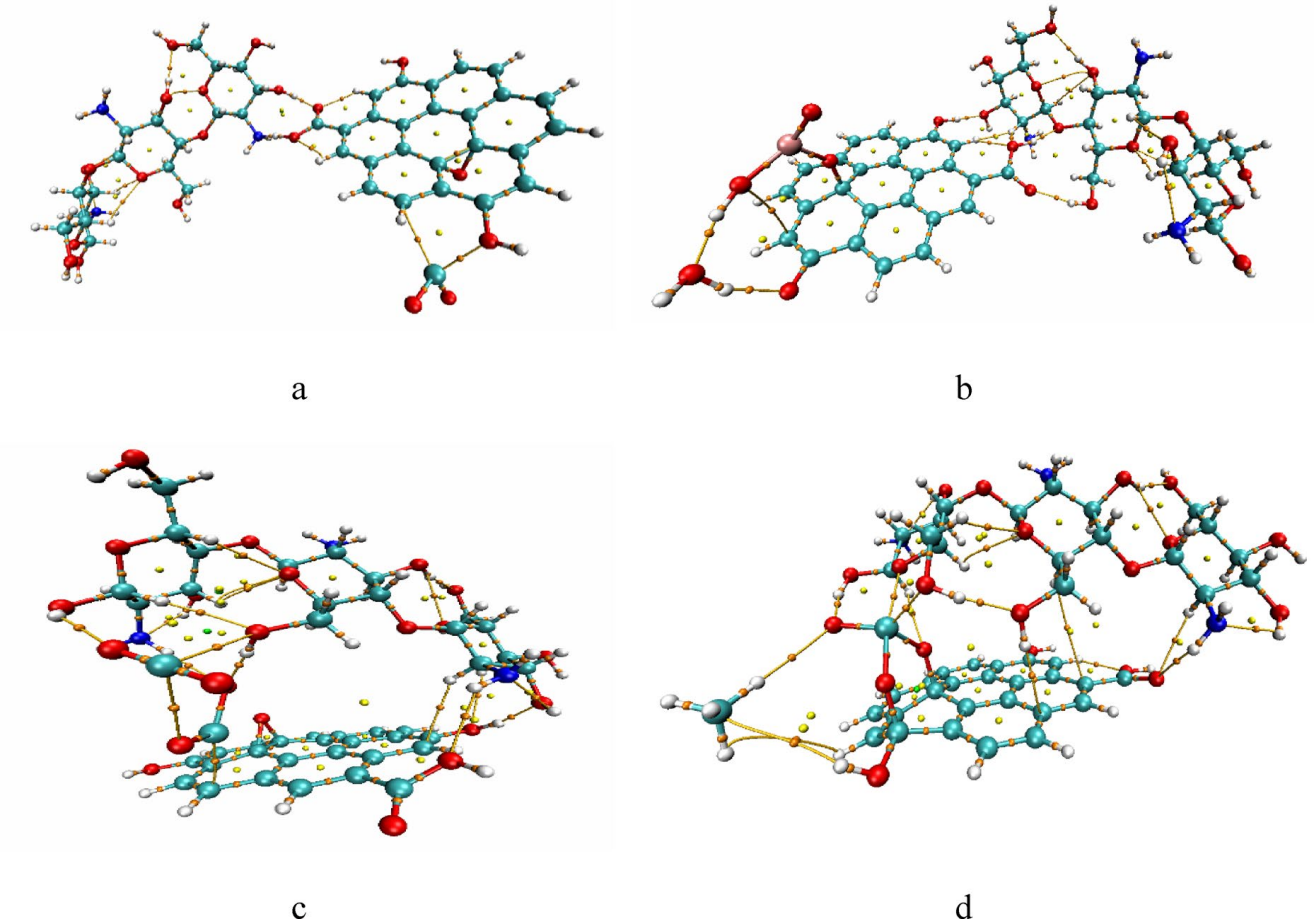
Calculated QTAIM topology for Cs/GO/TiO_2_ nanocomposite and the nanocompostie interacting with the gas molecules whereas; **a**- Cs/GO/TiO_2_ **b**- Cs/GO/TiO_2_/H_2_O, **c**- Cs/GO/TiO_2_/CO_2_ and **d**- Cs/GO/TiO_2_/CH_4_.

The Quantum Theory of Atoms in Molecules (QTAIM) provides a platform to analyze chemical bonding and intermolecular interaction, including non-covalent interactions. QTAIM utilizes electron density represented as ρ(r), which estimates the probability of an electron at any given point in space^52–54^. QTAIM maps ρ(r) and display regions of high and low electron density. By analyzing ρ(r), the Laplacian of electron density (∇²ρ) and the energy density H(r) at bond critical points (BCPs) and the type of bonding can be evaluated. Figures (11) illustrates the QTAIM topology for the nanocomposite and its interaction with the studied gases. The figure displaying the critical (CPs) and electron density paths, it shows the non-covalent interactions hydrogen bonding that stabilize the structures especially for figure (11-c) and (11-d). Upon investigating the BCPs between the Cs/GO/TiO_2_ and the gases, the results of ρ(r) < 0.2 a.u. and the positive values of ∇²ρ(r) and H(r) it indicates a weak interaction as proposed.

### Non-Covalent interaction (NCI)

**Fig. 12:**
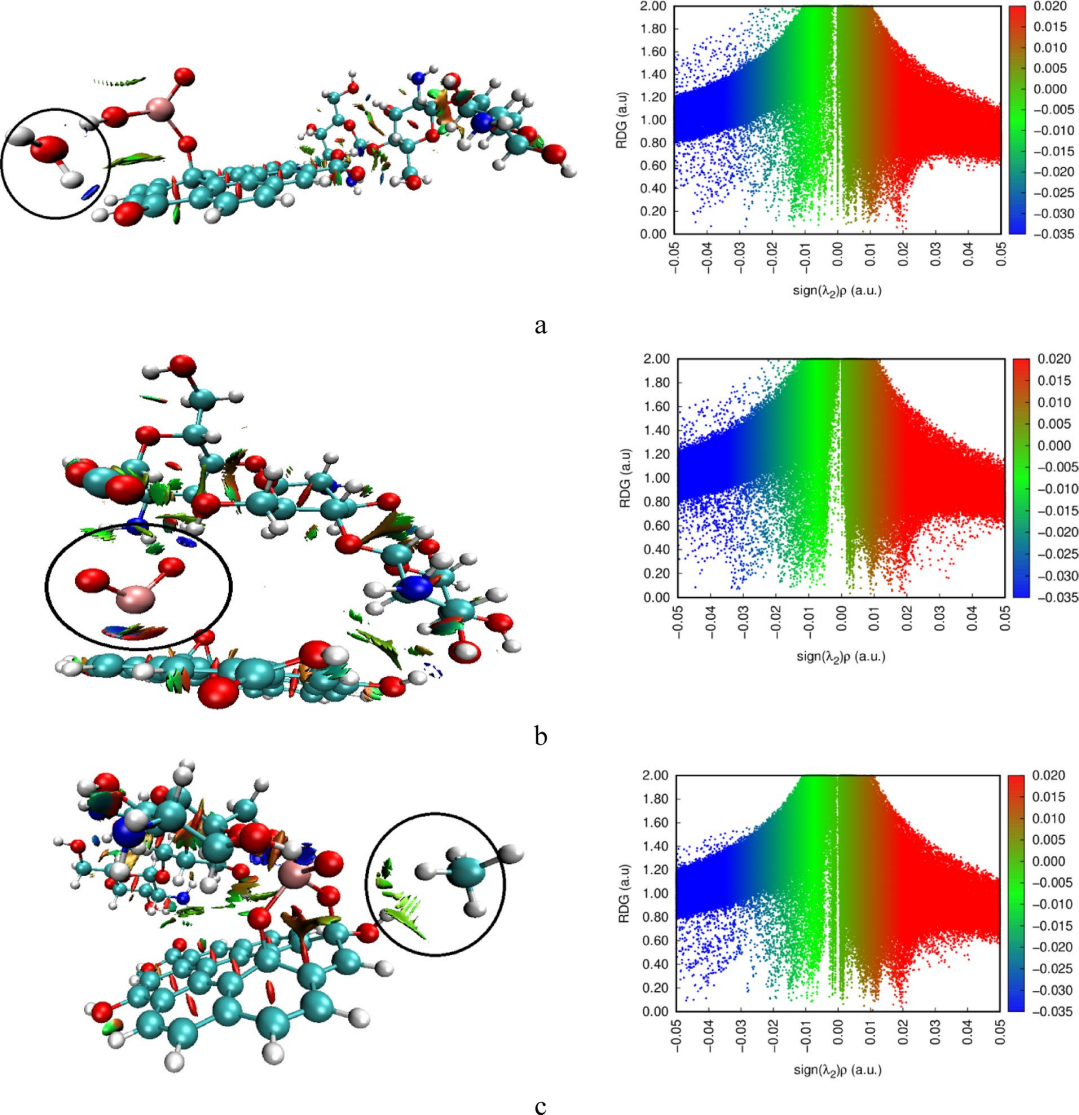
Calculated NCI for Cs/GO/TiO_2_ nanocomposite interacting with the gas molecules whereas; **a**- Cs/GO/TiO_2_/H_2_O, **b**- Cs/GO/TiO_2_/CO_2_ and **c**- Cs/GO/TiO_2_/CH_4_.

To gain deeper insight into the intermolecular interactions for the gas adsorbed complexes, non-covalent interaction (NCI) analysis complemented with reduced density gradient (RDG) plots. The calculated NCI and RDG plots at the same level of theory are presented in figure (12). NCI analysis visually represents non-covalent interactions, categorizing them as hydrogen bonds (blue), van der Waals (vdW) interactions (green), and steric repulsions (red). The weak interaction between GO, Cs and TiO_2_ in all complexes shows weak vdW attraction as indicated by green isosurfaces and spikes in RDG, contributing to its structural stability^55^. As for the gas and composite complexes, in figure (12- a), H₂O interacts with the composite via hydrogen bonding, as indicated by distinct blue isosurfaces. In figure (12-a), H_2_O interacts through hydrogen bond with the nanocomposite. In figure (12-b) CO_2_ interacts through weak van der Waals (green isosurfaces) and hydrogen/steric interactions (blue/red). For the interaction with CH_4_, figure (12-c) shows purely vdWs interactions with the nanocomposite to a greater extent. These findings are consistent with the results from the QTAIM analysis, which showed no covalent bond formation, and they align with the adsorption energy and Gibbs free energy values, confirming that all gas–composite interactions occur via physisorption - a desirable feature for sensor reversibility.

### Calculated IR spectra

**Fig. 13:**
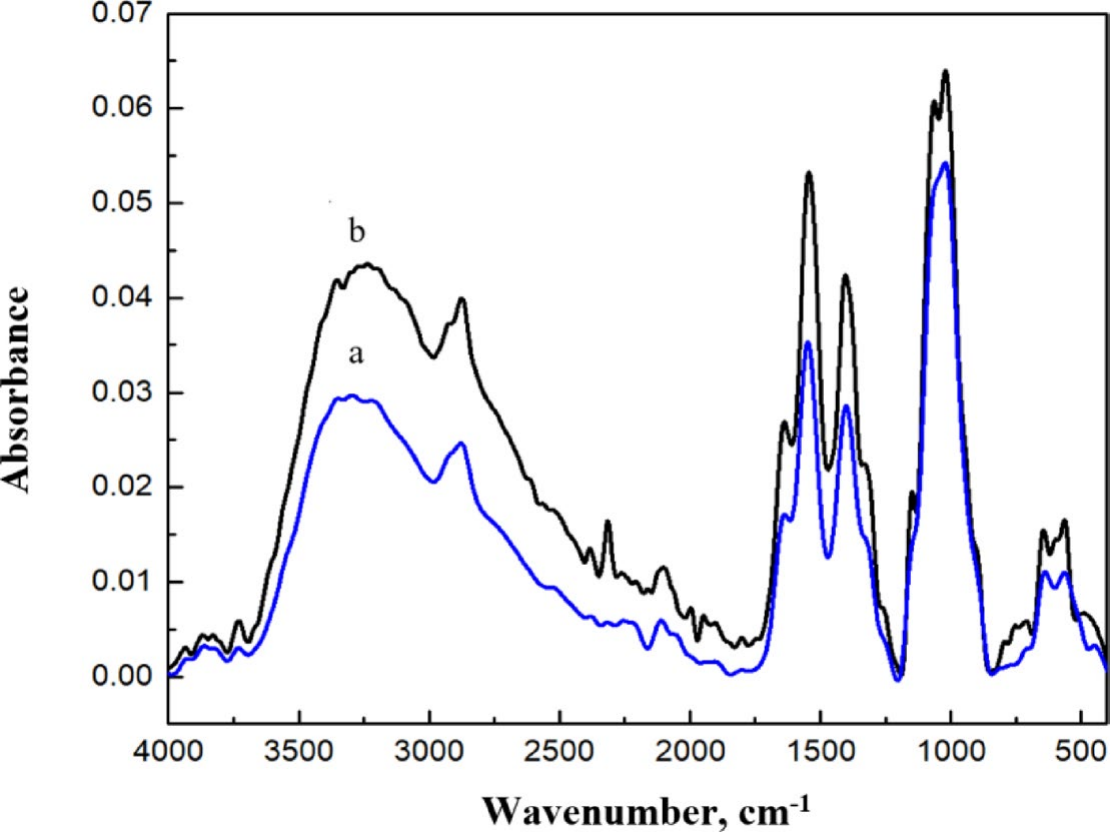
FTIR absorbance spectra for **a**- pure Cs and **b**-Cs/GO/TiO_2_ nanocomposite.

The infrared (IR) frequencies provide fingerprint information and can identify functional groups and other characteristic bands. Therefore, the IR frequencies of Cs/GO/TiO_2_ were calculated using DFT: B3LYP functional with LANL2DZ basis set and then compared to measured FTIR data. Figures (13) depicts the absorbance FTIR spectra for Cs/GO/TiO_2_ nanocomposite and the recorded band assignment in Table (5). For Cs IR spectra, the range 3350**–**3190 cm^−1^ is assigned to O–H and N–H stretching vibrations^56,57^. Symmetric/asymmetric CH_2_ stretching appears at 2930**–**2870 cm^−1^^54^. The C = O in amide I and N–H stretching (amide II) were observed at 1640 cm^−1^ and 1547 cm^−1^ respectively^58,59^. Additionally, stretching vibrations of carbodiimides CH_3_ stretching corresponds to 1403 cm^−1^^59^, and 1065 cm^−1^ to C–O stretching at^60,61^. For GO bands the O–H stretching appears at 3350**–**3190 cm^−1^^62^. At 1063 cm^−1^, the C–O epoxide stretching^62^, while the band C = C were observed at 1640 cm^−1^^62^. The C = O and O–C–O stretching appears at 1735 cm^−1^ and 1065 cm^−1^ respectively^60^. The band C–H stretching observed at 898 cm^−1^^63^. The characteristic band of Ti–O–Ti vibration falls within the range of 900**–**400 cm^−1^ were observed at 640**–**566 cm^−1^ and Ti–O stretching at 486 cm^−1^ within the same range^64,65^. Noticeably, the N–H stretching (amide II) band in the composite shifted from 1540 cm^−1^ (in pure Cs) to 1547 cm^−1^, indicating some physical interactions in the composite. Then the Computed IR frequencies were compared with FTIR measured results as in Table (6). The 3764**–**3705 cm^−1^ of O–H stretching corresponds to 3350**–**3190 cm^−1^ in FTIR while N–H stretching vibration appears at 3549**–**3455 cm^−1^ which correspond to the FTIR range of 3350**–**3190 cm^−1^. The CH_2_ stretching was found at 3137**–**3039 cm^−1^ match the experimental 2930**–**2870 cm^−1^. The N–H in amide II was found at 1597 cm^−1^ matching the 1547 cm^−1^. The C = C stretching corresponds to 1664**–**1639 cm^−1^ which match the FTIR of 1640 cm^−1^. The Ti–O stretching was found at 997**–**990 cm^−1^ matching the experimental value in the range 900**–**400 cm^−1^. The agreements between computed and experimental spectra validate the computational method.

**Table 5:** The band assignment of FTIR results for pure Cs and Cs/GO/TiO_2_ nanocomposite.

Structure	FTIR	Assignment	Ref
**Cs**	3350**–**3190	O–H N–H	56 57
	2930**–**2870	CH_2_	56
	1640	C = O in amide I	58
	1547	N–H amide II	59
	1403	CH_3_	58
	1065	C–O	60–61
**GO**	3350**–**3190	O–H	62
	1063	C–O epoxide	
	1640	C = C	
	1735	C = O	
	1065	O–C–O	
	898	C–H	63
**TiO**_**2**_	640**–**566	Ti–O–Ti	64
	486	Ti–O	65

**Table 6:** Computed IR frequencies compared with FTIR measured results for Cs/GO/TiO_2_.

Computed IR	FTIR		Assignment
3764 ~ 3705	3350**–**3190		O–H stretching
3549 ~ 3455	3350**–**3190		N–H stretching
3137 ~ 3039	2930**–**2870		CH_2_ stretching
1597	1547		N–H amide II
1664 ~ 1639	1640		C = C stretching
1574 ~ 1569	1735		C = O stretching
997 ~ 990	900**–**400		Ti–O stretching

## Conclusion

DFT: B3LYP/LANL2DZ was utilized to model Cs/GO/TiO₂ composite, then its interaction with H_2_O, CO_2_ and CH_4_ gases and studied their electronic properties. TDM, ΔE, global reactivity and MESP results indicated that the composite has the ability to interact with its surrounding molecules. The Cs/GO/TiO₂/CO₂ system exhibits optimal sensing characteristics, with a significantly reduced HOMO-LUMO energy gap (ΔE = 1.661 eV) and enhanced polarity (TDM = 22.229 Debye), indicating high sensitivity to environmental change. While for Cs/GO/TiO₂/H₂O (ΔE = 3.958 eV, TDM = 9.411 Debye) and Cs/GO/TiO₂/CH₄ (ΔE = 3.163 eV, TDM = 7.887 Debye) exhibit highest changes in energy gaps upon adsorption but show weaker TDM changes. HOMO/LUMO frontier orbitals, MESP, PDOS and Mulliken charge distribution confirmed electronic redistribution upon gas interaction. adsorption energy (Ea) and Gibbs free energy (ΔG) values revealed that CH₄ (Ea = 4.396 eV, ΔG = − 3.684 eV) and H₂O (Ea = 4.000 eV, ΔG = − 3.263 eV) are more thermodynamically favored, suggesting they are more suitable for practical sensing applications. QTAIM topology parameters (ρ(r), ∇²ρ(r), H(r)) and NCI plots demonstrated that all gas interactions occur via non-covalent, weak physical forces, which supports sensor reversibility and long-term reusability. Hydrogen and vdWs bonding within the composite’s internal structure also contributes to its overall stability. Cs/GO/TiO_2_ was prepared, and FTIR characterization confirmed the functional groups and then qualitatively compared them with computed IR for verification. Overall, Cs/GO/TiO₂ nanocomposite demonstrates strong potential as a multifunctional gas sensor, with high sensitivity and reversible sensing behavior — particularly toward CH₄ and H₂O under ambient conditions.

## Data Availability

The data supporting the findings of this study can be obtained from the corresponding author upon request, subject to reasonable conditions.
